# Large Vulvar Fibroepithelial Stromal Polyp With Superficial Vascular Thrombosis: A Case Report

**DOI:** 10.7759/cureus.102188

**Published:** 2026-01-24

**Authors:** Efthymia Thanasa, Emmanouil M Xydias, Anna Thanasa, Vasiliki Koutsia, Dimitra Koutsonikola, Konstantinos Zachos, Georgios Toulios, Apostolos C Ziogas, Ioannis Thanasas

**Affiliations:** 1 Department of Health Sciences, Medical School, Aristotle University of Thessaloniki, Thessaloniki, GRC; 2 Department of Obstetrics and Gynecology, EmbryoClinic IVF, Thessaloniki, GRC; 3 Department of Obstetrics and Gynecology, General Hospital of Trikala, Trikala, GRC; 4 Department of Surgery, General Hospital of Trikala, Trikala, GRC; 5 Department of Obstetrics and Gynecology, University of Thessaly, Larissa, GRC

**Keywords:** case report, clinical findings, histopathological diagnosis, surgical excision, vascular thrombosis, vulvar fibroepithelial stromal polyp

## Abstract

The present case report concerns a postmenopausal patient who presented to the gynecological outpatient clinic of the hospital with a two-year history of a pedunculated mass located in the vulvar region, which had gradually increased in size. During the preceding six months, the patient reported discomfort and difficulty during ambulation and sitting, as well as intermittent mild vulvar pain not associated with any local trauma. Clinical examination revealed a pedunculated vulvar lesion with a maximum diameter of approximately 45 mm, oval-shaped, semi-firm in consistency, and non-tender on palpation. Based on the clinical findings, a strong presumptive diagnosis of a vulvar fibroepithelial stromal polyp was made, and surgical excision of the lesion was decided. Histopathological examination of the surgical specimen confirmed the diagnosis of a vulvar fibroepithelial stromal polyp with superficial dermal vascular thrombosis. The postoperative course was uneventful. No evidence of disease recurrence was observed six months following surgery. The patient remains under regular follow-up with annual clinical evaluations to date. This case report highlights the unusual occurrence of a vulvar fibroepithelial stromal polyp with superficial vascular thrombosis in a postmenopausal patient without a history of hormone replacement therapy or tamoxifen use. Additionally, the main risk factors are reviewed, and the contemporary diagnostic approach to this rare pathological entity is discussed, emphasizing the pivotal role of surgical management and the necessity of distinguishing it from malignant vulvar conditions.

## Introduction

Fibroepithelial stromal polyps are benign, localized mesenchymal soft tissue lesions, the occurrence of which within the female genital tract may involve the vagina, the vulva, or the uterine cervix. Fibroepithelial stromal polyps most commonly arise in the vagina, whereas the vulva and the uterine cervix represent sites of less frequent involvement [1]. The first description of fibroepithelial polyps of the female genital tract in the English-language literature was provided by Norris and Taylor during the 1960s [2].

The vulvar fibroepithelial stromal polyp is a rare type of fibroblastic tumor arising in the vulvar region and is most commonly encountered in premenopausal women [3]. The size of vulvar fibroepithelial polyps varies considerably. Their minimum diameter may be only a few millimeters, whereas in rare cases they may reach a size exceeding 5 centimeters. In the majority of cases, as in our patient, the size of vulvar fibroepithelial polyps is less than 5 centimeters [4].

In the present case report, emphasis is placed on the unusual occurrence of a vulvar fibroepithelial stromal polyp with superficial dermal vascular thrombosis in a postmenopausal patient without a history of hormone replacement therapy or tamoxifen use. Furthermore, the main known risk factors are presented, and the contemporary diagnostic approach to this rare pathological entity is examined, highlighting the uniqueness of the surgical intervention and the necessity of differentiating it from malignant vulvar diseases.

## Case presentation

A 61-year-old patient with a history of four normal vaginal deliveries presented to the gynecological outpatient clinic of the General Hospital of Trikala, Greece, reporting that approximately two years earlier she had noticed the development of a pedunculated mass in the vulvar region, which had gradually increased in size. Additionally, during the preceding six months, she reported mild discomfort in the area of the external genitalia, as well as difficulty during ambulation and sitting. She also intermittently experienced mild vulvar pain, which was not associated with trauma to the vulvar area. The patient had been postmenopausal for the past 13 years. She reported a significant increase in body weight over the last decade (BMI = 32.3). Medical history revealed that she had not received hormone replacement therapy, nor had she been treated with tamoxifen at any point in her life. Her past medical history included hypothyroidism and arterial hypertension, both of which were well controlled with appropriate pharmacological treatment.

**Figure 1:**
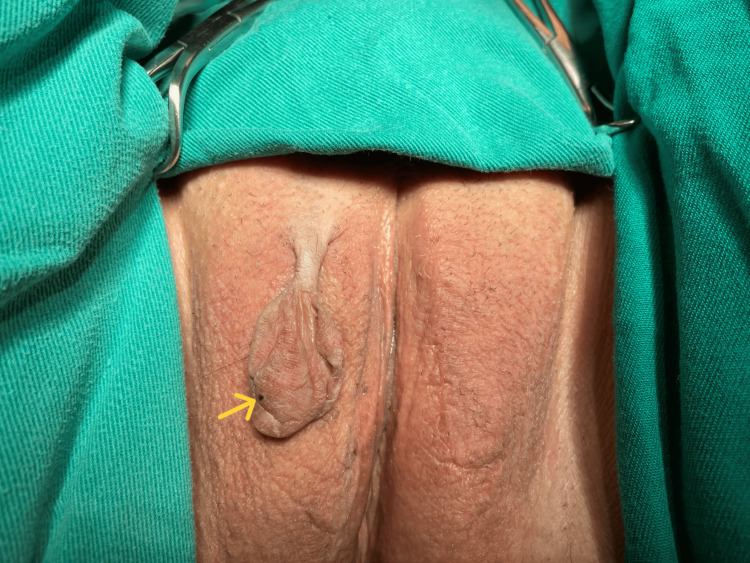
Patient in the gynecological position on the operating table prior to the initiation of surgery A pedunculated vulvar fibroepithelial stromal polyp with thrombosis of a superficial vessel is observed on the right labium majus (yellow arrow).

On inspection of the external genitalia, a pedunculated mass was identified in the upper third of the anterior surface of the right labium majus, oval in shape and measuring 45 × 27 × 10 mm, on the surface of which a small cyanotic lesion was visible (Figure 1).

The lesion was not surrounded by erythema and showed no macroscopic signs of inflammation. It was mobile, semi-firm in consistency, and non-tender on palpation. Preventive screening for cervical cancer, including conventional cytology and HPV DNA testing, yielded negative results. The results of the hematological examinations performed as part of the preoperative evaluation were within normal limits (Table 1).

**Table 1:** The preoperative laboratory tests of the patient Ht: hematocrit; Hb: hemoglobin; PLT: platelets; WBC: white blood cells; NEUT: neutrophils; APTT: activated partial thromboplastin time; INR: international normalized ratio; Glu: glucose; CEA: carcinoembryonic antigen; CA125: cancer antigen 125; CA15-3: cancer antigen 15-3; CA15-9: cancer antigen 19-9

Laboratory Tests	Preoperative Laboratory Tests	Reference Laboratory Tests
Ht	42.1%	37.7 – 49.7%
Hb	13.9 gr/dl	11.8 – 17.8 gr/dl
PLT	210x10^3^/ml	150 – 350 x10^3^/ml
WBC	7.85x40^3^/ml	4 – 10.8 x10^3^/ml
NEUT	51.4%	40 – 75%
APTT	26.1sec	24.0 – 35.0 sec
INR	0.91	0.8 – 1.2
Glu	91 mg/dL	75 – 115 mg/dL
CEA	2.37 ng/mL	< 5 ng/mL
CA125	17.9 U/mL	<= 35 U/mL
CA15-3	11.2 U/mL	0.0 – 31.3 U/mL
CA15-9	10.9 U/mL	0.0 – 37 U/mL

For the preoperative assessment of the vulvar lesion in our patient, the use of ultrasonography or magnetic resonance imaging was not considered necessary.

**Figure 2:**
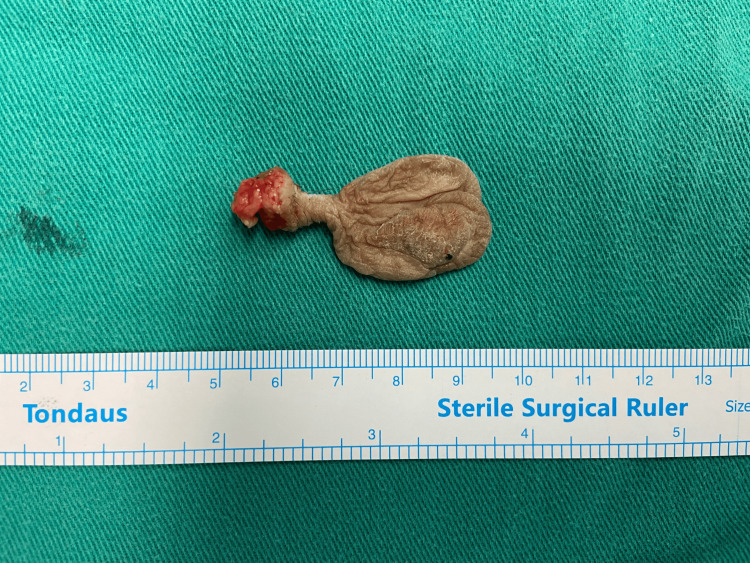
Surgical specimen of a vulvar fibroepithelial stromal polyp The tumor was excised with clear margins.

Based on the strong clinical suspicion of a vulvar fibroepithelial polyp, surgical excision of the vulvar lesion was decided. During surgery, a wide excision of the pedunculated mass with clear margins was performed (Figure 2).

**Figure 3:**
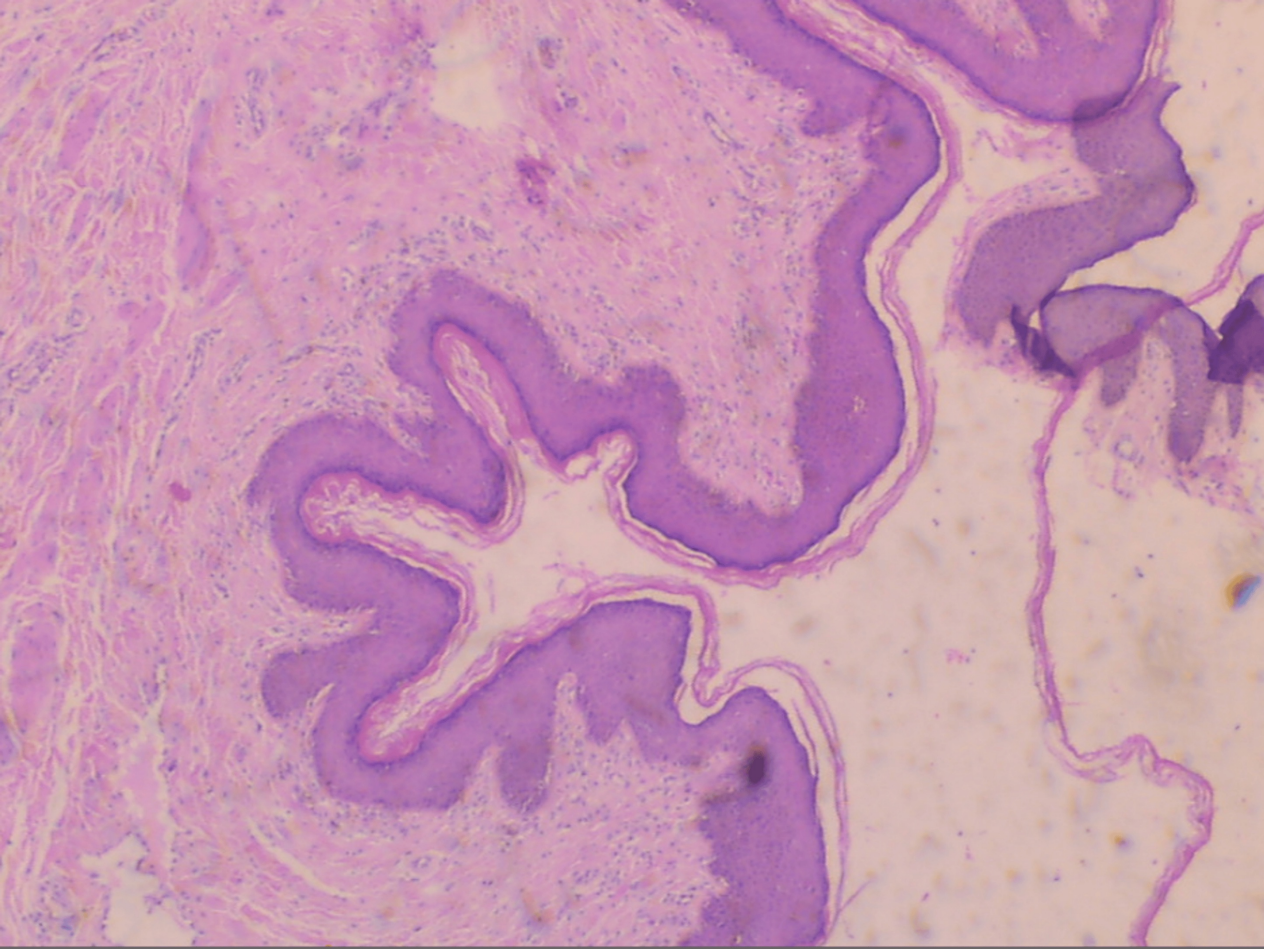
Histopathological image of a vulvar fibroepithelial stromal polyp A polypoid stromal vascular-rich lesion with collagen deposition and thrombosis of superficial dermal vessels is depicted (hematoxylin and eosin stain; magnification, ×10).

Histopathological examination of the surgical specimen confirmed the diagnosis of a vulvar fibroepithelial stromal polyp with thrombosis of superficial dermal vessels within the dermis. A polypoid vascular skin lesion extending to the epidermis with spindle and stellate cells without dysplastic epithelial changes was detected (Figure 3).

The postoperative course of our patient was uneventful. She was discharged from the gynecological ward on the morning of the following day. Six months after surgery, no recurrence was detected at the site of lesion excision. Follow-up of the patient at the gynecological outpatient clinic with regular annual visits is ongoing to date.

## Discussion

The pathogenesis of vulvar fibroepithelial stromal polyps has not yet been fully elucidated. Hormonal stimulation and chronic inflammatory processes have so far been proposed as the main risk factors. Vulvar fibroepithelial polyps are hormone-dependent lesions and are most commonly identified in women of reproductive age, in pregnant women, or in premenopausal women receiving hormone replacement therapy [5]. It is estimated that approximately 15% of vulvar fibroepithelial polyps occur during pregnancy; they are usually multiple and may occasionally regress spontaneously after childbirth [6]. Furthermore, the increased levels of hormones released during pregnancy are thought to regulate the growth of vulvar fibroepithelial polyps and to contribute to their enlargement. Immunohistochemical analysis has also demonstrated that vulvar fibroepithelial polyps are positive for vimentin, desmin, and estrogen and progesterone receptors, findings that support the hormone-dependent nature of these lesions [7]. In addition, vulvar fibroepithelial polyps are considered to occur more frequently in women undergoing hormone replacement therapy or in women receiving tamoxifen treatment [7]. Our patient was not pregnant, had not received hormone replacement therapy, nor had she been treated with tamoxifen at any point in her life. It is also noteworthy that our patient was postmenopausal. It is estimated that her increased body weight and chronic irritation in the genital region constituted the main contributing factors to the pathogenesis of the vulvar fibroepithelial polyp in this case [8]. The effect of obesity on the skin, unlike diabetes, cardiovascular diseases, and cancer, has not been well studied to date. It is estimated that obesity can cause skin manifestations, including fibroepithelial polyps [9].

The clinical diagnosis of vulvar fibroepithelial polyps is not always straightforward. The clinical presentations of vulvar lesions range from small papillomatous growths to large pedunculated masses. Vulvar fibroepithelial polyps are usually smaller than 5 centimeters [10]. The clinical features of these vulvar tumors are generally nonspecific. Small vulvar fibroepithelial polyps (<5 cm) are typically asymptomatic. In cases where they are symptomatic, common symptoms include bleeding, discharge from the mass, and mild discomfort due to the presence of the tumor in the vulvar region [7]. Pain is a clinical feature only in rare cases where vulvar fibroepithelial polyps reach large or giant dimensions [11]. Moreover, ulcerative and necrotic changes that may be observed on the surface of the tumor are associated with large polyps, which require careful differential diagnosis from malignant vulvar tumors [3,7].

Botryoid embryonal rhabdomyosarcoma and differentiated sarcomas constitute the main vulvar malignancies that must be differentiated from the benign vulvar fibroepithelial stromal polyp, although the differential diagnosis can be challenging [12]. Additionally, leiomyomas, superficial angiomyxomas, aggressive angiomyxomas, angiofibroblastomas, cellular angiofibromas, perineurinomas, and neurofibromas are other vulvar lesions that may mimic fibroepithelial polyps and should be considered in the differential diagnosis [13]. In our patient, the presence of a small thrombotic lesion on the surface of the tumor did not raise concern regarding malignancy. Histopathological examination of the surgical specimen confirmed the presence of a vulvar fibroepithelial stromal polyp with thrombosis of superficial dermal vessels and verified the benign nature of the lesion.

Significant assistance in the preoperative diagnosis of large vulvar fibroepithelial polyps can be provided by ultrasonography and magnetic resonance imaging. Ultrasonographic examination can be considered the first-line diagnostic approach due to its favorable cost-effectiveness, wide availability, and capability for dynamic assessment compared with magnetic resonance imaging [14]. Pelvic magnetic resonance imaging is able to demonstrate a large, solid, irregular mass with a polypoid contour and exophytic extension into the vulva, with or without the presence of a stalk and thickening of the polypoid wall. The presence of inguinal lymphadenopathy is indicative of malignancy of the vulvar lesion [15]. In general, although the findings of magnetic resonance imaging of vulvar fibroepithelial polyps are often similar to those of aggressive angiomyxoma, angiofibroblastoma, and cellular angiofibroma, the presence of stromal hypointense regions surrounded by perilous areas on T2-weighted MRI and hyperintense areas on T1-weighted MRI supports the diagnosis of a fibroepithelial stromal polyp [16]. In our patient, the use of imaging studies was deemed unnecessary. The preoperative diagnosis was based on clinical findings. Wide surgical excision of the lesion was selected as the most appropriate therapeutic option.

Biopsy and histopathological examination followed by wide local excision with clear margins of the vulvar lesion constitute the treatment of choice for large and giant vulvar fibroepithelial stromal polyps [17]. In cases where the polyp measures only a few millimeters, management may be limited to cryotherapy or local cauterization of the tumor. The disadvantage of both of these therapeutic approaches is that they do not provide material for histopathological diagnosis [18]. Accurate histopathological diagnosis is essential for the effective management of vulvar fibroepithelial polyps and for their differentiation from malignant vulvar lesions [3].

The prognosis is favorable. Recurrence of vulvar fibroepithelial polyps is rare, particularly in cases treated with wide local excision with clear margins. Concurrently, long-term follow-up to monitor for potential local recurrence should be considered an essential recommendation for every patient [15]. In our patient, histopathological examination confirmed the diagnosis of a vulvar fibroepithelial stromal polyp and excluded the presence of atypia or malignancy in the vulva. Regular follow-up with annual visits to the gynecological outpatient clinic of the General Hospital of Trikala was recommended. Six months after surgical excision of the polyp, no local recurrence was observed in our patient. 

## Conclusions

Fibroepithelial stromal polyps are rare benign tumors of the female genital tract. The occurrence of large vulvar fibroepithelial polyps with thrombosis of superficial dermal vessels, as observed in our patient, is even more uncommon. The association between obesity in menopause and the appearance of the tumor may be possible. In any case, however, more studies that will substantiate this hypothesis in the future are considered necessary.

## References

[1] Schoolmeester JK, Fritchie KJ (2015). Genital soft tissue tumors. J Cutan Pathol.

[2] Norris HJ, Taylor HB (1966). Polyps of the vagina. A benign lesion resembling sarcoma botryoides. Cancer.

[3] Bahadur A, Mundhra R, Heda A, Singh A (2024). Large vulvar fibroepithelial polyp and review of differentials. BMJ Case Rep.

[4] Lozano-Peña AK, Lamadrid-Zertuche AC, Ocampo-Candiani J (2019). Giant fibroepithelial polyp of the vulva. Australas J Dermatol.

[5] Hartmann CA, Sperling M, Stein H (1990). So-called fibroepithelial polyps of the vagina exhibiting an unusual but uniform antigen profile characterized by expression of desmin and steroid hormone receptors but no muscle-specific actin or macrophage markers. Am J Clin Pathol.

[6] Pearl ML, Crombleholme WR, Green JR, Bottles K (1991). Fibroepithelial polyps of the vagina in pregnancy. Am J Perinatol.

[7] Madueke-Laveaux OS, Gogoi R, Stoner G (2013). Giant fibroepithelial stromal polyp of the vulva: largest case reported. Ann Surg Innov Res.

[8] Navada MH, Bhat PR, Rao SV, G N (2011). Large fibroepithelial polyp of vulva. Case Rep Dermatol Med.

[9] Lau K, Höger PH (2013). Skin diseases associated with obesity in children (Article in German). Bundesgesundheitsblatt Gesundheitsforschung Gesundheitsschutz.

[10] Lawal BK, Yahya A, Zubair SY, Abubakar M, Kabir B, Oguntayo AO, Kolawole AO (2024). Giant fibro-epithelial polyp of the vulva: a case report. J West Afr Coll Surg.

[11] Shehata F, Benzaquen S (2023). Giant vulvar fibroepithelial stromal polyp. J Obstet Gynaecol Can.

[12] Amin A, Amin Z, Al Farsi AR (2018). Septic presentation of a giant fibroepithelial polyp of the vulva. BMJ Case Rep.

[13] Raphael Avidime A, Usman H (2017). Bilateral giant fibroepithelial labial mass: a case report. J Obstet Gynaecol Can.

[14] Testa AC, Fruscella E, Ludovisi M (2005). The role of sonographic examination in the follow-up of gynecological neoplasms. Gynecol Oncol.

[15] Tripathi PU, Suryarao P, Patvekar MM, Kolte D (2024). Unveiling the truth: fibroepithelial polyp of the vulva and its misdiagnosis as cancer. Cureus.

[16] Kato H, Kanematsu M, Sato E, Ito N, Furui T, Hirose Y (2010). Magnetic resonance imaging findings of fibroepithelial polyp of the vulva: radiological-pathological correlation. Jpn J Radiol.

[17] Mishra A, Pandey RK (2016). Fibro-epithelial polyps in children: a report of two cases with a literature review. Intractable Rare Dis Res.

[18] Orosz Z, Lehoczky O, Szoke J, Pulay T (2005). Recurrent giant fibroepithelial stromal polyp of the vulva associated with congenital lymphedema. Gynecol Oncol.

